# What determines plant species diversity along the Modern Silk Road in the east?

**DOI:** 10.1002/imt2.74

**Published:** 2023-01-09

**Authors:** Yanlei Liu, Chao Xu, Wenpan Dong, Xun Chen, Wen Zhang, Yuzhe Sun, Guohong Wang, Yufei Wang, Shiliang Zhou

**Affiliations:** ^1^ School of Landscape and Ecological Engineering Hebei University of Engineering Handan China; ^2^ State Key Laboratory of Systematic and Evolutionary Botany, Institute of Botany Chinese Academy of Sciences Beijing China; ^3^ College of Life Science University of Chinese Academy of Sciences Beijing China; ^4^ Laboratory of Systematic Evolution and Biogeography of Woody Plants, School of Ecology and Nature Conservation Beijing Forestry University Beijing China; ^5^ Hulunbuir University Hulunbuir China; ^6^ State Key Laboratory of Vegetation and Environmental Change, Institute of Botany Chinese Academy of Sciences Beijing China

**Keywords:** desert area, DNA metabarcoding, environmental DNA, plant species diversity, Silk Road

## Abstract

As primary producers, plants provide food, oxygen, and other resources for global ecosystems, and should therefore be given priority in biodiversity protection. Most biodiversity research focuses on biodiversity hotspots, while biodiversity coldspots, such as deserts, are largely ignored. We propose that the factors shaping plant species diversity differ between biodiversity hot spots and cold spots, especially for desert ecosystems. To test this hypothesis, we investigated plant species diversity along the Modern Silk Road in the Northwest China desert, an area characterized by low precipitation, scarce vegetation, a limited number of species, and variable human activities. Surface soil was sampled from 144 plots, environmental DNA (eDNA) was extracted from soil samples, and seed plant species were identified using DNA metabarcoding technology. A total of 671 seed plant species were detected, which was more diverse than indicated by plot survey data. Plant species diversity gradually decreased from east to west along the Silk Road. In this area, temperature determines plant species diversity more than precipitation. Additionally, human activity has altered plant species diversity by introducing crops and invasive plants and eliminating environmentally adapted indigenous plants. Our results demonstrate the potential of eDNA metabarcoding technology for plant species diversity surveying. Desert plants have adapted to dry environments by relying on underground water or utilizing occasional rainfall as ephemerals, which are often not visible during surface surveys because of their short aboveground life cycle but can be detected with eDNA metabarcoding technology. Groundwater maintenance and human activity control are recommended for plant species diversity conservation and desertification control.

## Highlights

- Environmental DNA (eDNA) is a quick and accurate method for biodiversity assessment.
- This study first used eDNA‐assessed regional plant species diversity.
- Temperature instead of precipitation is the main factors affecting plant diversity.
- Human activities were proven to affect plant species diversity in desert areas.
- More instant plant diversity results need to be done for desertification control.

## Graphical abstract

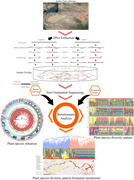

## INTRODUCTION

Biodiversity is a measure of Earth's vitality and the basis for human survival and development. Hence, protecting biodiversity is akin to protecting humankind. As primary producers, plants provide food, oxygen, and other resources for global ecosystems, and should therefore be given priority in biodiversity protection. The conventional method of investigating plant species diversity is surface plant surveys, which require extensive sampling throughout the year and can be expensive, time‐consuming, and labor‐intensive. Additionally, correct specimen identification can be a challenge due to a shortage of taxonomists [1] and species with minor or tiny bodies are often ignored [2]. The recent advent of DNA barcoding technology [3, 4], an environmental DNA (eDNA)‐based method, makes rapid identification of species in environmental samples possible [2, 5, 6]. eDNA is extracted from samples on the premise that an environmental sample, such as soil, water, feces, or air, may contain remains and free DNA of plants, animals, and microorganisms. DNA metabarcoding is an application of the next‐generation sequencing (NGS) platform‐based metagenomics technology [2, 3, 6, 7]. A mixture of DNA from different species can be sequenced, with the resulting sequences identified to species using DNA barcoding.

DNA metabarcoding of environmental samples has been used in paleobiodiversity reconstruction [8, 9, 10, 11], modern biodiversity assessment [12, 13], biological monitoring [14], invasive species assessment [11], animal feeding habit assessment [15], plant and microorganism interaction research [16], and forensic evidence analysis [17]. While some review articles mention using environmental samples for plant diversity research, eDNA has not yet been extensively used to study plant species diversity on a large geographical scale. Plants are ideal candidates for eDNA research because most plant species have been documented more than animals and microorganisms. Seed plant species in China have been compiled in “Flora Reipublicae Popularis Sinicae” and “Flora of China,” and the reference library of “DNA Barcodes of Plants in China” has been substantially constructed. The main challenge with DNA barcoding lies in collecting representative soil samples. When studying current plant species diversity, sampling surface soil to a depth no greater than 5 cm provides plant information about several decades. Most previous metabarcoding research was related to animals and microorganisms, for which sampling strategies differ from plants. Unlike plants, animals have a large range of motion, and therefore require deeper soil samples. For microorganisms, their large copy numbers make it possible to obtain a large amount of microbial information with a small amount of soil, preventing sampling depth from becoming a limitation. Plants store their genetic information in the form of DNA or DNA carriers in the corresponding soil stratum at different time periods. The deeper the bottom layer, the older the plant information obtained [18]. Although research on plant species diversity based on topsoil is rare, previous research indicates that the eDNA in topsoil can reflect modern plant information since the diversity of plant species obtained from soil was richer than that growing above ground [18].

Most studies on species diversity have focused on biodiversity “hotspots.” Desert areas are characterized by scarce precipitation, poor soil conditions, and harsh ecological environments with limited biodiversity, known as biodiversity “coldspots.” Biodiversity coldspots generally have a few plant species, most of which are sensitive to environmental changes and human activities [19, 20]. The world is currently experiencing coldspot expansion and hotspot contraction due to widespread desertification [21]. The Eurasian desert, the world's second‐largest desert, covers an area stretching from central China to the east coast of the Mediterranean Sea, coincident with the Silk Road. The Northwest China desert is located in the eastern part of the Silk Road, an area that has witnessed the rise and fall of many ancient civilizations and once played a key role in the economic and cultural exchange between the east and the west. Previous plant community surveys of the area indicated poor species diversity and plant communities characterized by small ephemeral herbs and drought‐resistant herbs, shrubs, and small trees [20, 22].

In this study, we will use DNA metabarcoding technology to explore plant species in the Northwest China desert to test the hypotheses that (1) plant species diversity is greater than indicated by plant community surveys, and (2) the factors shaping geographical plant diversity patterns differ between biodiversity hotspots and coldspots. Desert areas frequently have strong winds that disperse plant litter relatively evenly. Therefore, collections of surface soil and plant litter represent local flora better than plant community surveys. Species that have recently completed their aboveground life cycle or disappeared from an area are likely to deposit detectable plant remains in the topsoil. Species identified by DNA metabarcoding are easier and more accurate for situations lacking diagnostic characteristics of certain organs, such as for deserts where the flora tends to be relatively simple. Desert flora are the result of evolution and adaption to local environmental factors, such as precipitation, temperature, soil, and human activity. The factors contributing most to biodiversity loss, as well as how to effectively conserve desert plant species diversity, remain to be explored.

In this study, we collected topsoil samples, meteorological records, and human population sizes of the nearest villages from 144 plots along the eastern Silk Road in the Northwest China desert to answer the following questions: (1) what are the spatial patterns of seed plant species diversity? (2) which factors contribute most to these spatial patterns; and (3) how have human activities affected the local plant species diversity? Our aim is that the results will improve our understanding of plant species diversity in the Northwest China desert and contribute to the development of strategies to conserve biodiversity, halt desertification, and restore desert vegetation.

## METHODS

### Study site and soil sample collection

**Figure 1:**
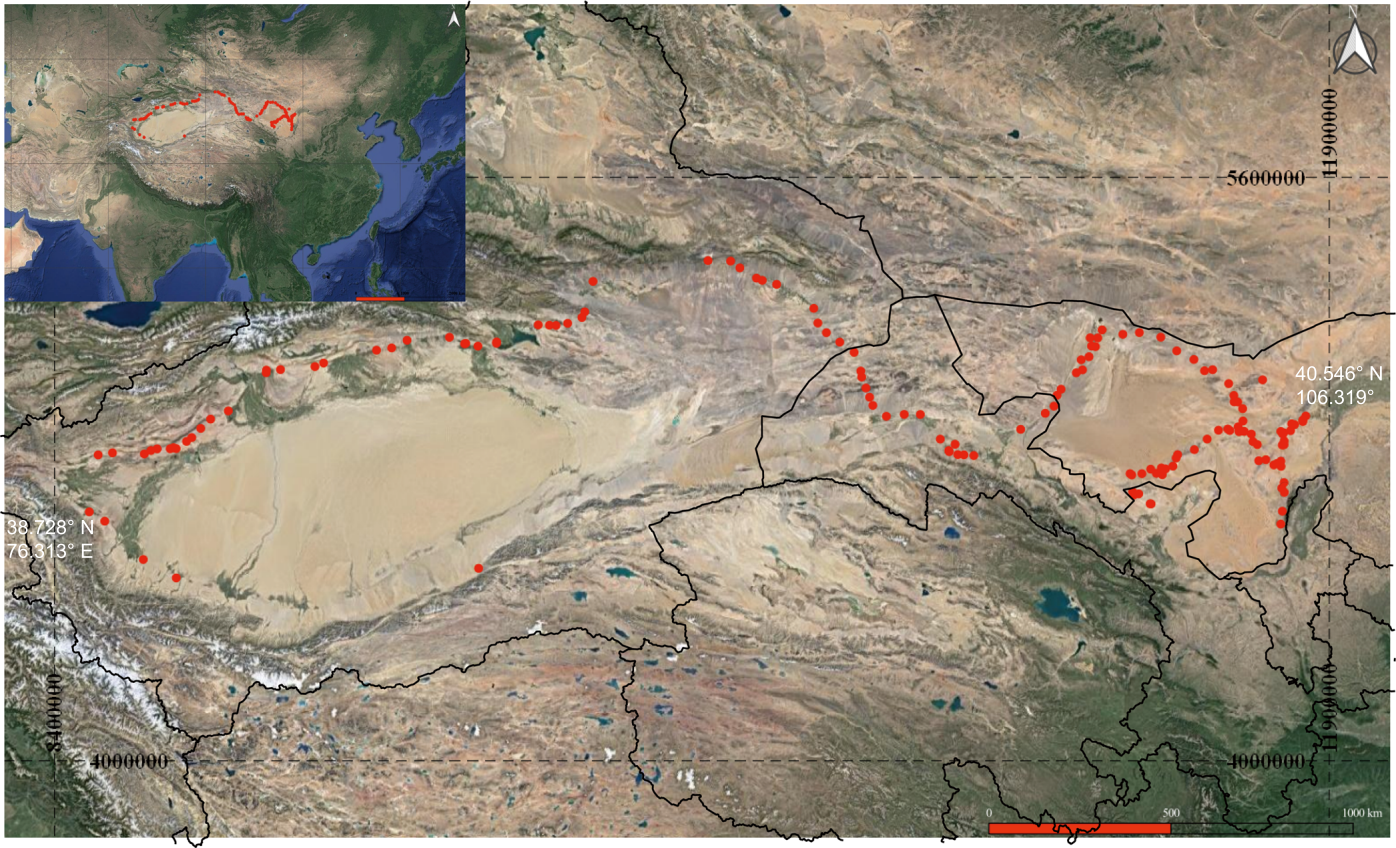
Surface soil sample collection sites in the northwest desert area along the ancient Silk Road in China. Sampling sites were marked using the red dot according to their location information.

Sampling ranged place from June 8 to July 21, 2015. The field area ranged over 3600 km eastward–westward in the Northwest China Desert, from Alxa Left Banner, Inner Mongolia (40.546° N, 106.319° E) on the easternmost side to Yingjisha, Kashi City, Xinjiang Province (38.728° N, 76.313° E) on the westernmost side (Figure 1). The vegetation of sampled areas belonged to three desert vegetation zones: temperate shrubby steppe, temperate shrubby and semishrubby, and warm temperate shrubby and semishrubby (Table S1). Environmental factors varied significantly from site to site (Table S1). The average annual rainfall ranged from 221 mm in Inner Mongolia to 30 mm in the middle of the Tarim basin. Approximately half of the annual precipitation in these areas occurs during the summer in China. The average annual temperature ranges from 5.2°C to 12.4°C, and the average monthly temperature ranges from 18.1°C to 28.5°C during the summer and −10.7°C to −2.5°C during the winter. The human population density ranges from 0.166 to 84.707 people per square kilometer. According to Species2000 and Global Biodiversity Information Facility, 1559 seed plant species belonging to 504 genera of 87 families (Table S2) grow in this area, which serves as an upper limit for DNA metabarcoding of the sequences in this area.

Five topsoil samples (5 cm deep) were collected from four corners and one in center (the standard five‐spot‐sampling method) in a 1000 cm × 1000 cm quadrat for each plot. The five soil samples were equally polled and dried in an oven at 65°C for 24 h to prevent DNA from degrading and kept at −20°C until DNA extraction.

### Ecological data collection

The plant species diversity in soil samples is a random sample of the plant species diversity in recent a few decades [23]. Therefore, the averages over the last 30 years were used as climate indicators. Climate indicators for each plot, including the average annual temperature, monthly temperature, seasonal temperature, annual precipitation, monthly precipitation, and seasonal precipitation of the last 30 years, were downloaded from WorldClim (http://www.worldclim.org) using locality information of each sampling plot. Sampling sites belonging city human population density, major human settlements (above township level, including township level), and dryness (annual evaporation and drought index) were also collected from the website of each related town. Surface plant species were recorded when sampling the surface soil which only represents the current year's plant diversity (Table S1).

### Soil DNA extraction

Before DNA extraction, we conducted experiments on the extraction of Soil DNA with PowerSoil DNA extraction kit and the traditional modified cetyltrimethylammonium bromide (mCTAB) method [24] (Figure S1). The results show that both the mCTAB method and the PowerSoil kit have obtained eDNA with obvious main bands, and both have the same performance in the amplification experiments of rbcL1 universal fragments. The recovery efficiency of the kit meets the needs and saves time. After several pilot studies on DNA extraction, we finally selected PowerSoil DNA extraction kit (Thermo Fisher Scientific) to extract DNA from the 144 surface soil samples in this study. Ten grams of well‐mixed soil in each sample were grounded into fine powder using a mortar and pestle. We followed the DNA extraction instructions given by the kit manufacturer. Three DNA extractions per sample were performed to lower the possibility of random failure and the resultant DNA from the same sample was combined. Negative controls were conducted using DNA‐free water to detect potential lab DNA pollution.

### PCR amplification

Due to the relatively rich species coverage in the public database, three plant‐specific chloroplast fragments, *rbcL*, *matK*, and *trnL‐*intron, were used as DNA barcodes in this study. To meet the read length requirement of the Illumina platform, intermediate primers were used to amplify *matK* and *rbcL* fragments of approximately 400 bp. The primer pairs used for fragment amplification were rbcLbF and rbcL717LR for *rbcL* [25], matK472F and matK821R for *matK* [26], and trnLc and trnLh for *trnL*‐intron [27]. The Illumina platform was used to sequence the amplicons according to the methods of Liu et al. [28]. Each primer was labeled at the 5′‐end by 8 bp unique oligoes of 24 kinds (Table S1) for discriminating fragments from different samples in a sequencing library. The PCR procedures and programs with the unique oligo‐labeled primer pairs were the same as Dong et al. [29]. To reduce PCR bias, the same amplification was repeated three times and the three products were combined for subsequent treatment. The PCR products were checked by 1.5% agarose gel electrophoresis.

### NGS library construction and sequencing

For each DNA barcode, the PCR products of every 24 samples labeled with different oligoes were combined together, forming a sequencing library. There are six groups in total mixed separately and purified using the Promega Wizard DNA Clean‐Up System (Promega). After quantification with a Qubit fluorescence quantifier (Thermo Fisher Scientific), to reduce sequencing libraries, the purified PCR products of *matK* and *rbcL* were pooled at nearly equal molar amounts for the consideration that they are of similar lengths. A total of 12 sequencing libraries (six for *matK* + *rbcL*, and six for *trnL*‐intron) were constructed and sequenced at Beijing Novogene Co. Ltd. on the Illumina platform. The sequencing modes were PE250 for *matK* + *rbcL* and PE150 for *trnL*‐intron (Table S3).

### DNA barcode‐reference library construction

For accurate assignment of species names to the sequences by DNA barcoding, all the *rbcL*, *matK*, and *trnL*‐intron sequences of all seed plants in Northwest China [30] were acquired from the reference library of Barcode of Plant in China (State Key Laboratory of Systematic and Evolutionary Botany, Institute of Botany, Chinese Academy of Sciences, unpublished). Three local DNA barcode‐reference libraries were constructed according to the DNA barcodes.

### NGS data processing

The data analysis methods and tools used were the same as Liu et al. [28]. The quality control of the raw data from the Illumina platform was carried out using the NGS QC toolkit v2.3.3 [31] with the default parameters. The read1 and read2 were merged using PANDAseq v2.11 [32] with the default parameters. The sequences from the same library were sorted into *matK* and *rbcL* according to the primer sequences with fqgrep v0.4.4 (https://github.com/indraniel/fqgrep) and further assigned to each sample according to the unique oligoes using FASTX v0.0.13 toolkit software (http://hannonlab.cshl.edu/fastx_toolkit/).

### Data analysis

#### Feature table preparation

A feature table is a summary table containing the read number of plant species in each sample. The feature table was constructed by mapping the original reads of each sample to the reference data using a 0.99 similarity in Usearch v10.0.240 (https://drive5.com/downloads/usearch10.0.240_win32.exe.gz) for further analysis. Since three barcodes were used in this study, three feature tables were created. The feature tables were standardized using Usearch v10.0.240 by adjusting each sample read number to 10,000. Due to a lack of a reliable DNA reference library of lower plants, only seed plants were used in this study. The three feature tables were merged according to the data ratio of angiosperms and gymnosperms (i.e., *matK* primers were majorly amplified for angiosperms while *rbcL* and *trnL*‐intron primers were majorly amplified for seed plants, including angiosperms and gymnosperms). Thus, the *rbcL* and *trnL*‐intron feature tables were merged equally first and the read number was adjusted to 10,000 for each sample. Then, the newly produced feature table was merged with the *matK* feature table according to the proportion of angiosperms.

#### Plant species distribution patterns

The plant species in all samples were summarized using Microsoft Excel2016 and visualized using GraPhlAn v1.1.3 [33]. The plant species distribution pattern in the area was summarized using Microsoft Excel2016 based on the species detected in each sampling site (=each sample) and visualized using Adobe Illustrator CC 2018. A plant species diversity map was pictured by QGIS v3.6.3‐Noosa to understand the plant species distribution trend in the area.

#### Association between plant species diversity and its potential driving elements

Associations between potential driving factors and native plant species diversity (Table S1) were tested using R v4.2.1. The correlation analyses were conducted between climate factors and the alpha diversity indexes obtained from the data set of wild plant species (Table S1). Climate factors included temperature, precipitation, and other related factors, and the alpha diversity indexes included Shannon.e diversity diversity, Chao1 diversity, Berger Parker diversity, Buzas Gibson diversity, Dominance index, Equitability diversity, Jost diversity, and Richness and Simpson diversity. A correlation analysis was also conducted between the results of plant species obtained from soil DNA and the surface plant species using the R v4.2.1 based on alpha diversity indexes obtained from the Usearch v10.0.240 alpha_div command. The results of all correlation analyses are visualized using Cytoscape v3.9.1 [34].

#### Unweighted pair‐group method with arithmetic (UPGMA) means analysis between plant species diversity and areas of major human settlement

Associations between human population density and native plant species diversity (Table S1) were tested using R v4.2.1. The correlation analyses were conducted between the alpha diversity indexes obtained from the data set of wild plant species and climate factors (Table S1).

## RESULTS

### DNA barcode‐reference library

The DNA barcode‐reference library consisted of three data sets: *rbcL*, *matK*, and *trnL*‐intron. The *rbcL* data set consisted of 853 species in 361 genera of 80 families, while *matK* consisted of 944 species in 374 genera of 81 families and *trnL*‐intron consisted of 686 species in 316 genera of 70 families. In total, the three DNA barcode‐reference libraries consisted of 1183 species in 432 genera of 83 families, which accounted for 76% of the species, 86% of the genera, and 95% of the families in the area.

### Seed plant species detected from soil samples

**Figure 2:**
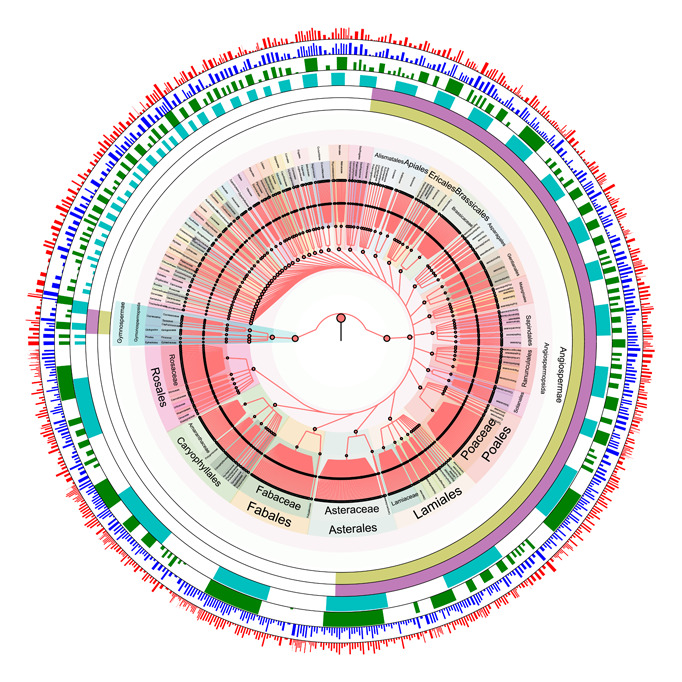
General situation of plant species in the desert area of northwest China. Statistics of relative species richness at the level of species (red), genera (dark blue), families (green), orders (light blue), classes (pink), and phylum (yellow) represented by the outer inner rings (from outside to inside), the value is the logarithm of sequence number divided by 10 in each species.

In total, 3.02, 2.29, and 19.08 million clean reads were collected from the Illumina platform for *rbcL*, *matK*, and *trnL*‐intron, respectively. After mapping clean reads to reference data sets, 671 seed plant species in 291 genera of 65 families were detected from the 144 surface soil samples (Table S4 and Figure 2). Among these, 623 were wild plant species in 266 genera of 62 families (Figure S2), 20 were invasive species, and 28 were cultivated species. According to the number of reads, which is a rough estimate of the abundance of individuals, the top five most abundant families were Asteraceae (16.11%), Rosaceae (12.50%), Pinaceae (6.73%), Nitrariaceae (6.09%), and Fabaceae (5.94%) (Figure S3A). The top five most abundant genera were Artemisia (7.73%), Picea (6.68%), Nitraria (5.87%), Juniperus (4.36%), and Ulmus (4.34%) (Figure S3B). The top 10 most frequently observed species were *Nitraria sibirica* (2.63%), *Iljinia regelii* (2.30%), *Ulmus glaucescens* (2.17%), *Ulmus pumila* (2.17%), *Picea asperata* (1.67%), *Picea crassifolia* (1.67%), *Picea obovate* (1.67%), *Picea schrenkiana* (1.67%), *Typha angustifolia* (1.29%), and *Morus nigra* (1.28%) (Figure S3C). The five richest families (by a number of species) were Poaceae (14.13%), Asteraceae (12.20%), Rosaceae (10.91%), Fabaceae (7.06%), and Ranunculaceae (5.62%) (Figure S3D).

### Seed plant species observed in surface quadrats

Through the surface vegetation investigation, 87 plant species were identified, belonging to 59 genera of 23 families. Amaranthaceae (20 species), Fabaceae (13 species), Asteraceae (13 species), Poaceae (eight species), and Zygophyllaceae (six species) were the top five species‐rich families. Artemisia (five species), Caragana (five species), Salsola (five species), and Zygophyllum (four species) were the species‐richest genera (Table S5). Among these genera, 44 species were found in the soil samples, representing 50.6% of plant species obtained in surface quadrats.

### Spatial patterns of species diversity

**Figure 3:**
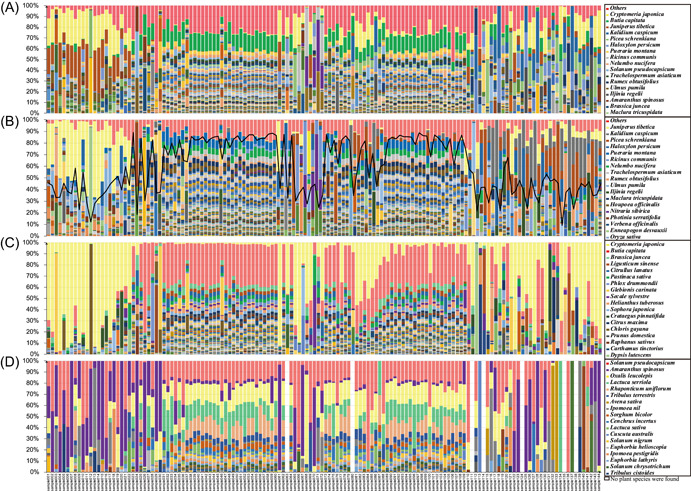
The plant species diversity distribution pattern in the northwest desert area of China based on plant species composition in each sampling site. (A) All plants, (B) wild plants, (C) cultivated plants, and (D) invasive plants.

The geographical pattern of plant species diversity was outlined using the composition of seed plants from each sampled site, including native wild, cultivated, and invasive species. The number and relative richness of plant species in the middle region were more complex than those in the outer regions. The composition of plant species in the middle region was in a balanced state, without dominant species from current decades (Figure 3A). For example, there were 383 plant species at Site 040, with the largest proportion of 3.14% for *Vitis vinifera*. In contrast, *Enneapogon desvauxii* showed a 29.46% dominance at Site 012 in the east and *I. regelii* showed a 25.68% dominance at Site 137 in the west. The Shannon.e diversity index showed a similar trend for native wild (Figure 3B), cultivated, and invasive species (Figure 3C,D). Plant species diversity generally decreased from east to west (Figure 3). The largest exception to this trend was a group of 20 sites in the east (in the Ulan Buhe and Tengger deserts) that exhibited lower plant species diversity because they contain hardly any vegetation.

### Association between plant species diversity and ecological factors

**Figure 4:**
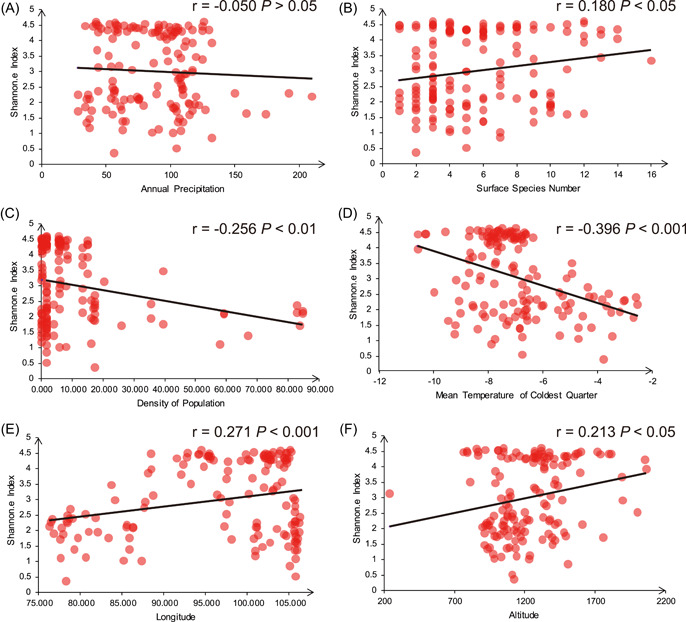
Correlation analysis between Shannon.e diversity Index and potential affecting factors. Shannon Index correlation results with (A) annual precipitation, (B) surface species number, (C) density of population, (D) mean temperature of coldest quarter, (E) longitude, and (F) altitude.

**Figure 5:**
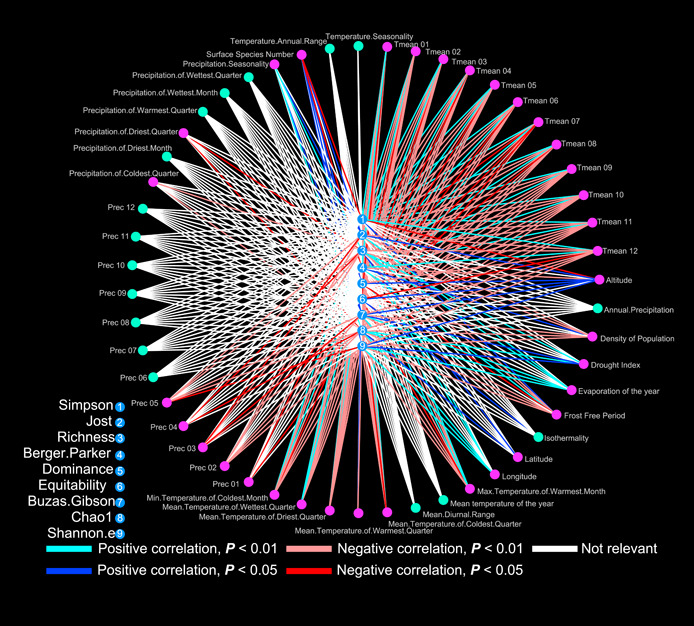
Correlation analysis results of each diversity index. Lines with different colors are used to express whether the results of correlation analysis are statistically significant.

The Shannon.e diversity index was positively correlated with altitude and longitude (*P* < 0.05), and negatively correlated with annual temperature over the last 30 years, the average temperature from January to December, the maximum temperature in the warmest month, the average temperature in the coldest month, the average temperature in the coldest season, the average temperature in the warmest season, and the frost‐free period (Table S6, and Figures 4 and 5). The Chao1 diversity index was positively correlated with latitude, longitude, and average precipitation in each season (*P* < 0.05) and negatively correlated with temperature (*P* < 0.05) (Table S6, Figure 5, and Figure S4). Correlations between the other alpha diversity indexes and factors exhibited similar tendencies as the Shannon diversity and the Chao1 diversity indexes (Table S6, and Figure 5).

### Association between plant species diversity and human population size

**Figure 6:**
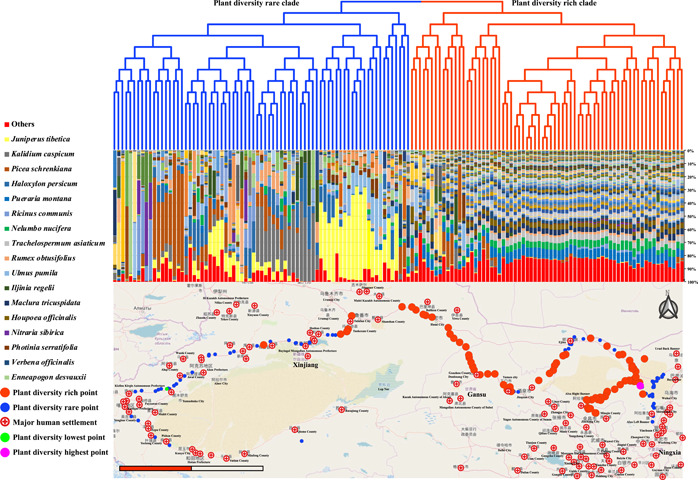
UPGMA clustering map based on the plant species diversity in the desert area. The dendrogram and plant composition heat map were obtained based on UPGMA clustering of wild plant species composition in each sampling site. The plant species diversity situation and major human settlements were marked on the map (red dots represent plant biodiversity rich point; small blue dots represent plant biodiversity rare point; red plus symbols with a circle represent the major human settlements). UPGMA, unweighted pair‐group method with arithmetic.

Population size reflects human interference to the environment. The population size and the plant species diversity index were significantly negatively correlated (Table S6, and Figure 5). The UPGMA dendrograms showed an association between plant species diversity and human population size, with less native plant species diversity in areas with more human settlements (Figure 6). For example, site 023 had the maximum Shannon.e diversity index of 4.61 (purple circle in Figure 6), while site 134 had the minimum Shannon.e diversity index of 0.359 (green circle in Figure 6). These results imply that human activities are related to the decline of plant species diversity in the Northwest China desert.

## DISCUSSION

### Environmental sampling is a quick, accurate, and cost‐effective method

Surface plant surveys were previously the only practical way to evaluate plant species diversity [20, 22, 35] but were expensive, time‐consuming, and labor‐intensive. Now, DNA metabarcoding of environmental samples is a viable alternative. A direct comparison of this study with a traditional quadrat survey in the same area [36, 37] reached similar conclusions on plant species composition, the local dominant species, and the geographical patterns of species diversity based on more reliable data (Figure 3).

A sharp discrepancy between this and previous studies is the number of species detected: 671 versus 87, respectively. The number of species detected in this study is more reasonable due to the large sampling area and species diversity present. Plant organs, such as pollen grains, can be carried into a plot from outside their boundaries by wind or water and can only be detected with eDNA metabarcoding. Ephemeral plants that appear and disappear from plots are identifiable based on their remains by eDNA metabarcoding. While environmental plant DNA metabarcoding can be powerful, our method only identified approximately half (50.6%) the number of plant species observed in traditional surveys. Similar research was rarely conducted until now, with most current research focusing on certain groups and constructing a reference database based on the group, in contrast to our study. Large debris was removed from the samples before soil grinding to reduce DNA bias, which is likely the cause of this difference. Additionally, our study compared the diversity of aboveground and environmental samples and found no direct correlation between the two diversities. The surface plants represent plant species diversity in the sampling year, while the environmental samples represent the plant species diversity of recent decades, making it inappropriate to compare the information gathered from the two methods. The number of species found on the soil surface is small and random, which is also different from the traditional method of conducting large‐scale and long‐term surface plant surveys. The zonal statistics of surface plant information based on 1 year alone cannot reflect the current situation of plant species diversity in this area. Moreover, among the 43 species missed with eDNA metabarcoding, 19 are in Amaranthaceae (formerly Chenopodiaceae), seven in Asteraceae, and five in Fabaceae. DNA barcoding has a downside of low resolution for closely related species when using conventional chloroplast fragments, so the sequences of the closely related undetected species may have been treated as background noise during data processing. The internal transcribed spacer (ITS) between the small and large subunits of the ribosomal RNA gene works well for species discrimination, but eDNA often contains a large number of microorganisms and the plant ITS sequences could be overwhelmed by the ITS sequence of microorganisms.

Compared with the traditional plot survey method, eDNA metabarcoding has several advantages: ease of use, speed, labor‐savings, unlimited sampling time, and no need for prior taxonomic knowledge. eDNA metabarcoding is especially useful in the dynamic monitoring of plant species diversity over a large geographical scale in a short time [2, 5, 12, 38]. The maturity of DNA labeling of samples [28] also makes it affordable for a large number of samples. Mixed sequencing of multiple samples using the amplicon sequence of labeling PCR markers will save the cost of the original NGS library building, library inspection, and redundant sequences, reducing the cost to one‐tenth of the original. Moreover, a large amount of amplicon data can be obtained with limited funds, which can help revitalize this field.

### Effects of ecological factors on plant species diversity

According to environmental cybernetics, climate, physical, and other ecological factors are the dominant forces in the formation of plant species diversity patterns on multiple scales [39, 40]. Water and energy (mainly temperature) are the two most important environmental factors affecting plant diversity [41, 42]. In this study, precipitation was surprisingly unrelated to plant species diversity. Unlike moist, tropical, and subtropical areas [13, 43, 44, 45, 46], the precipitation in desert areas is too scarce to play an important role in the formation of plant species diversity, with desert plants relying mainly on underground water. Counterintuitively, there was a tendency of annual mean temperature decrease from northwest to southeast in the sampled areas. Plant species diversity was negatively correlated with the temperature in the Northwest China desert, which was first observed by [20, 35] based on data from traditional surveying. A possible mechanism for this trend is that higher temperatures induce more water evaporation that aggravates aridity and lowers plant species diversity.

The association of plant population localities by latitude, longitude, and altitude to plant species diversity is the outcome of factors, such as energy, water (especially underground water), and soil [47]. Latitude was not significantly correlated with plant species diversity in this study, probably due to the small range of latitudes among sampling sites (standard deviation = 1.2°). Longitude was significantly positively correlated with plant species diversity, although the impact of precipitation seemed irrelevant. Owing to the high elevation in the west, water (especially the underground water from mountain glaciers) flows eastward, as with the Tarim River (the longest inland river in China). The communities in the east enjoy more underground water supplies [48] and maintain higher plant species diversity. Plant species diversity decreased with increasing altitude due to the reduction of species and their distribution evenness, confirming the findings of [49]. To understand which factors contributed most to the formation of geographical patterns of plant species diversity, we conducted principal component analysis (PCA) of 26 elements based on the Shannnon.e diversity index. There were 26 significantly related factors based on the Shannnon.e diversity index. The top 16 influencing factors were temperature related, indicating the importance of temperature in this desert area. Longitude was the second most influencing factor (Table S7).

### Human activities affect plant species diversity

Human population growth and the increasing scope and intensity of human activities have caused habitat loss and fragmentation that threaten the survival and reproduction of species. Human population size increased along the sampling belt from east to west and was negatively correlated with plant species diversity (Figures 4 and 5, and Table S6). Human activity was the third major factor affecting plant species diversity in PCA results, indicating the impact of human activity on local species diversity (Table S7). Because habitat restoration is more difficult in sensitive and fragile desert ecosystems, restriction of human activities, such as overexploitation and farming, is the simplest way to protect desert ecosystems and maintain their ecological authenticity.

### Desert plant species diversity conservation

Deserts are dry, hot in summer, and cold in winter, with desert plants adapted to limited habitats in these conditions. Plants that manage well in desert areas of the Northwest include xerophytes, such as Zygophyllum spp., Haloxylon spp., and Calligonum spp.; phreatophytes, such as *Atriplex patens* (Litv.) Iljin, *Alhagi sparsifolia* Shap., and *Tamarix chinensis* Lour.; and ephemerals, such as *Erodium oxyrrhynchum* M. Bieb., *Alyssum liniolium* Steph. ex Willd., and *Schismus arabicus* Nees. However, parasites, such as *Cynomorium songaricum* Rupr. and *Cistanche deserticola* Ma, rely on trees and shrubs. Many desert plant species are endemic, such as *Ammopiptanthus mongolicus* (Maxim. ex Kom.) Cheng f., *Potaninia mongolica* Maxim., Pugionium spp., *Stilpnolepis centiflora* (Maxim.) Krasch., *S. intricata* (Franchet) C. Shih., *Tetraena mongolica* Maxim., and *Tugarinovia mongolica Iljin*. They are considered relict flora of the Paleomediterranean components from the Tertiary due to the uplift of the Himalayas [50]. Many desert‐endemic plants are used as herbal medicines of high economic value. For example, *Arnebia euchroma* (Royle) Johnst. is used in the treatment of measles, constipation, burns, frostbite, eczema, and dermatitis, while *C. songaricum* and *C. deserticola* are used as aphrodisiacs. Many of these economically valuable desert plants have been listed as endangered species due to overexploitation.

Compared with biodiversity hotspots, desert plant species diversity is low and vegetation coverage is poor. Ground vegetation takes a long time to establish and can suffer from instant destruction. The negative correlation between species diversity and human population size indicates that human activities in the Northwest desert have contributed to the loss of desert plant species diversity (Figures 4 and 5). Additionally, more heavily populated areas experienced more invasive species and imbalance of native species, warranting urgent conservation of both quantity of vegetation and the number of plant species.

eDNA and metabarcoding work well for the monitoring of dynamic changes of native plant species diversity and invasive plants that are essential for effective conservation. Metabarcoding has been successfully used on microorganisms [13, 51] and animals [9, 11], as well as plants in this study. Our results will promote plant species diversity protection via rapid and accurate evaluation of large‐scale plant species, establishment of national desert parks or nature reserves, and domestication economically valuable species. Creating desert nature reserves would be unlikely to restrict human activities in most of the Northwest China desert. Rather, a few areas of high desert plant species diversity would be protected as national parks or nature reserves, in which human activities could be managed and other endangered desert plants could be introduced for conservation. Domesticating species of economic value, such as *A. euchroma*, would aid conservation by allowing for cultivation and preventing foraging from fragile ecosystems. As parasites, *C. songaricum* and *C. deserticola* are more difficult examples but still hold potential.

## CONCLUSION

The study revealed spatial patterns of plant species diversity based on species identified from eDNA extracted from 144 topsoil samples collected along the Silk Road in the Northwest China desert. In all, 671 plant species were detected, suggesting that the plant species diversity is not as low as indicated by previous plant community surveys. The species predominantly belonged to Asteraceae, Rosaceae, Poaceae, Pinaceae, Nitrariaceae, and Fabaceae. Plant species diversity was higher in the east than in the west and was more affected by temperature than precipitation. Underground water is more vital to the survival of desert plants, and maintenance of a high underground water level is essential for preventing desertification. Human activities profoundly impact plant species diversity by reducing native wild species and introducing crops and invasive plants. Dynamic monitoring of desert plant species diversity using eDNA metabarcoding technology is advised for conservation efforts. Domestication of economic plants is one possible solution to discourage local people from overexploiting natural resources.

## AUTHOR CONTRIBUTIONS

**Shiliang Zhou**: Conceptualization; methodology; writing – review and editing; supervision; funding acquisition. **Yanlei Liu**: Conceptualization; methodology; formal analysis; writing – original draft; writing – review and editing. **Chao Xu**: Methodology; formal analysis; investigation; writing – review and editing; funding acquisition. **Yufei Wang**: Conceptualization; methodology; funding acquisition. **Wenpan Dong**: Methodology; formal analysis. **Xun Chen**: Formal analysis. **Wen Zhang**: Formal analysis. **Yuzhe Sun**: Formal analysis. **Guohong Wang**: Investigation. All authors have read and approved the final manuscript.

## CONFLICT OF INTEREST

The authors declare no conflict of interest.

## Supporting information

Supporting Information

Table S1. Sampling situations, including physical sampling conditions, alpha diversity results and ecological factors information of each sampling site. Table S2. A list of plant species in the desert region of Northwest China. Table S3. NGS library situation constructed in this study. Table S4. Feature table of plant species obtained by DNA metabarcoding. Table S5. A list of plant species obtained from a plant surface survey based on relative density. Table S6. Correlation analysis results between alpha diversity and ecological factors.Table S7. PCA analysis results of ecological factors significantly related to Shannon.e.

## Data Availability

NGS merged original data from Illumina Hiseq2500 platforms are submitted to NCBI and the accession numbers are SRR19552682 for *matK* gene; SRR19553404 for *matK* gene's barcode; SRR19553109 for *rbcL* gene; SRR19552957 for *rbcL* gene's barcode; SRR19553105 for *trnL‐F* intron and SRR19553403 for *trnL‐F* intron's barcode. Supplementary materials (figures, tables, scripts, graphical abstract, slides, videos, Chinese translated version, and update materials) may be found in the online DOI or iMeta Science http://www.imeta.science/

## References

[1] Yang, Qiner . 2001. “Over‐Reliance of SCI Damages the Research of Traditional Taxonomy in China—Some Thoughts After Reading Two Letters in “Nature”.” Journal of Systematics and Evolution 39: 283. https://www.jse.ac.cn/EN/Y2001/V39/I3/283

[2] Taberlet, Pierre , Eric Coissac , François Pompanon , Christian Brochmann , and Eske Willerslev . 2012. “Towards Next‐Generation Biodiversity Assessment Using DNA Metabarcoding.” Molecular Ecology 21: 2045–50. 10.1111/j.1365-294X.2012.05470.x 22486824

[3] Hebert, Paul D. N. , Sujeevan Ratnasingham , and Jeremy R. de Waard . 2003. “Barcoding Animal Life: Cytochrome *c* Oxidase Subunit 1 Divergences Among Closely Related Species.” Proceedings of the Royal Society of London. Series B: Biological Sciences 270: S96–9. 10.1098/rsbl.2003.0025 PMC1698023 12952648

[4] Kress, W. John . 2017. “Plant DNA Barcodes: Applications Today and in the Future: Plant DNA Barcode Applications.” Journal of Systematics and Evolution 55: 291–307. 10.1111/jse.12254

[5] Bohmann, Kristine , Alice Evans , M. Thomas P. Gilbert , Gary R. Carvalho , Simon Creer , Michael Knapp , Douglas W. Yu , and Mark de Bruyn . 2014. “Environmental DNA for Wildlife Biology and Biodiversity Monitoring.” Trends in Ecology & Evolution 29: 358–67. 10.1016/j.tree.2014.04.003 24821515

[6] Deiner, Kristy , Holly M. Bik , Elvira Mächler , Mathew Seymour , Anaïs Lacoursière‐Roussel , Florian Altermatt , Simon Creer , et al. 2017. “Environmental DNA Metabarcoding: Transforming How We Survey Animal and Plant Communities.” Molecular Ecology 26: 5872–95. 10.1111/mec.14350 28921802

[7] Mardis, Elaine R . 2008. “Next‐Generation DNA Sequencing Methods.” Annual Review of Genomics and Human Genetics 9: 387–402. 10.1146/annurev.genom.9.081307.164359 18576944

[8] Bremond, Laurent , Charly Favier , Gentile Francesco Ficetola , M. G. Tossou , A. Akouégninou , Ludovic Gielly , C. Giguet‐Covex , Richard Oslisly , and U. Salzmann . 2017. “Five Thousand Years of Tropical Lake Sediment DNA Records from Benin.” Quaternary Science Reviews 170: 203–11. 10.1016/j.quascirev.2017.06.025

[9] Willerslev, Eske , John Davison , Mari Moora , Martin Zobel , Eric Coissac , Mary E. Edwards , Eline D. Lorenzen , et al. 2014. “Fifty Thousand Years of Arctic Vegetation and Megafaunal Diet.” Nature 506: 47–51. 10.1038/nature12921 24499916

[10] Wiens, John J. , and Michael J. Donoghue . 2004. “Historical Biogeography, Ecology and Species Richness.” Trends in Ecology & Evolution 19: 639–44. 10.1016/j.tree.2004.09.011 16701326

[11] Ficetola, Gentile Francesco , Jérôme Poulenard , Pierre Sabatier , Erwan Messager , Ludovic Gielly , Anouk Leloup , David Etienne , et al. 2018. “DNA from Lake Sediments Reveals Long‐Term Ecosystem Changes After a Biological Invasion.” Science Advances 4: eaar4292. 10.1126/sciadv.aar4292 29750197 PMC5942909

[12] Yu, Douglas W. , Yinqiu Ji , Brent C. Emerson , Xiaoyang Wang , Chengxi Ye , Chunyan Yang , and Zhaoli Ding . 2012. “Biodiversity Soup: Metabarcoding of Arthropods for Rapid Biodiversity Assessment and Biomonitoring.” Methods in Ecology and Evolution 3: 613–23. 10.1111/j.2041-210X.2012.00198.x

[13] Tedersoo, Leho , Mohammad Bahram , Sergei Põlme , Urmas Kõljalg , Nourou S. Yorou , Ravi Wijesundera , Luis Villarreal Ruiz , et al. 2014. “Global Diversity and Geography of Soil Fungi.” Science 346: 1256688. 10.1126/science.1256688 25430773

[14] Thomsen, Philip Francis , and Eske Willerslev . 2015. “Environmental DNA—An Emerging Tool in Conservation for Monitoring Past and Present Biodiversity.” Biological Conservation 183: 4–18. 10.1016/j.biocon.2014.11.019

[15] Berry, Tina E. , Sylvia K. Osterrieder , Dáithí C. Murray , Megan L. Coghlan , Anthony J. Richardson , Alicia K. Grealy , Michael Stat , Lars Bejder , and Michael Bunce . 2017. “DNA Metabarcoding for Diet Analysis and Biodiversity: A Case Study Using the Endangered Australian Sea Lion (*Neophoca cinerea*).” Ecology and Evolution 7: 5435–53. 10.1002/ece3.3123 28770080 PMC5528208

[16] Ding, Xia , Feng Jin , Jiawang Xu , Shulei Zhang , Dongxu Chen , Beijuan Hu , and Yijiang Hong . 2022. “The Impact of Aquaculture System on the Microbiome and Gut Metabolome of Juvenile Chinese Softshell Turtle (*Pelodiscus sinensis*).” iMeta 1: e17. 10.1002/imt2.17 PMC10989827 38868566

[17] Liu, Yanlei , Chao Xu , Wenpan Dong , Xueying Yang , and Shiliang Zhou . 2021. “Determination of a Criminal Suspect Using Environmental Plant DNA Metabarcoding Technology.” Forensic Science International 324: 110828. 10.1016/j.forsciint.2021.110828 34000616

[18] Edwards, Mary E. , Inger Greve Alsos , Nigel Yoccoz , Eric Coissac , Tomasz Goslar , Ludovic Gielly , James Haile , et al. 2018. “Metabarcoding of Modern Soil DNA Gives a Highly Local Vegetation Signal in Svalbard Tundra.” The Holocene 28: 2006–16. 10.1177/0959683618798095

[19] Dang, Rongli , Xiaoling Pang , and Xuefeng Gu . 2002. “Floristic Analysis of Spermatophyte Genera in the Arid Deserts Area in North‐West China.” Guihaia 22: 8. http://qikan.cqvip.com/Qikan/Article/Detail?id=6209879

[20] Wang, Jianming , Wenjuan Wang , Jingwen Li , Yiming Feng , Bo Wu , and Qi Lu . 2017. “Biogeographic Patterns and Environmental Interpretation of Plant Species Richness in Desert Regions of Northwest China.” Biodiversity Science 25: 1192–201. 10.17520/biods.2017149

[21] Bouquet, Christian . 2017. “Le Sahara entre ses deux rives. Éléments de délimitation par la géohistoire d'un espace de contraintes.” Géoconfluences 1: 1–15. http://geoconfluences.ens-lyon.fr/informations-scientifiques/dossiers-regionaux/afrique-dynamiques-regionales/articles-scientifiques/sahara-entre-deux-rives

[22] Lu, Kaiqing , Feng Qin , Yang Li , Gan Xie , Jinfeng Li , Yiming Cui , David K. Ferguson , et al. 2020. “A New Approach to Interpret Vegetation and Changes Through Time by Establishing a Correlation Between Surface Pollen and Vegetation Types in the Eastern Central Asian Desert.” Palaeogeography, Palaeoclimatology, Palaeoecology 551: 109762. 10.1016/j.palaeo.2020.109762

[23] Yoccoz, G. Nigel , Kari Anne Bråthen , Ludovic Gielly , James Haile , Mary E. Edwards , Tomasz Goslar , H. Von Stedingk , et al. 2012. “DNA from Soil Mirrors Plant Taxonomic and Growth Form Diversity.” Molecular Ecology 21: 3647–55. 10.1111/j.1365-294X.2012.05545.x 22507540

[24] Li, Li , Wang Wang , Yu Yu , Wang Wang , and Zhou Zhou . 2013. “A Modified CTAB Protocol for Plant DNA Extraction: A Modified CTAB Protocol for Plant DNA Extraction.” Chinese Bulletin of Botany 48: 72–78. 10.3724/SP.J.1259.2013.00072

[25] Dong, Wenpan , Tao Cheng , Changhao Li , Chao Xu , Ping Long , Chumming Chen , and Shiliang Zhou . 2014. “Discriminating Plants Using the DNA Barcode *rbcL*b: An Appraisal Based on a Large Data Set.” Molecular Ecology Resources 14: 336–43. 10.1111/1755-0998.12185 24119263

[26] Yu, Jing , Jianhua Xue , and Shiliang Zhou . 2011. “New Universal *matK* Primers for DNA Barcoding Angiosperms.” Journal of Systematics and Evolution 49: 176–81. 10.1111/j.1759-6831.2011.00134.x

[27] Taberlet, Pierre , Eric Coissac , Francois Pompanon , Ludovic Gielly , Christian Miquel , Alice Valentini , Thierry Vermat , et al. 2007. “Power and Limitations of the Chloroplast *trn* L (UAA) Intron for Plant DNA Barcoding.” Nucleic Acids Research 35: e14. 10.1093/nar/gkl938 17169982 PMC1807943

[28] Liu, Yanlei , Chao Xu , Yuzhe Sun , Xun Chen , Wenpan Dong , Xueying Yang , and Shiliang Zhou . 2021. “Method for Quick DNA Barcode Reference Library Construction.” Ecology and Evolution 11: 11627–38. 10.1002/ece3.7788 34522329 PMC8427591

[29] Dong, Wenpan , Chao Xu , Changhao Li , Jiahui Sun , Yunjuan Zuo , Shuo Shi , Tao Cheng , Junjie Guo , and Shiliang Zhou . 2015. “ycf1, the Most Promising Plastid DNA Barcode of Land Plants.” Scientific Reports 5: 8348. 10.1038/srep08348 25672218 PMC4325322

[30] Liu, Bing . 2022. “China Checklist of Higher Plants.” In Catalogue of Life China: 2022 Annual Checklist, Biodiversity Committee of Chinese Academy of Sciences, ed. Beijing: Catalogue of Life China. http://www.sp2000.org.cn/info/info_how_to_cite

[31] Patel, Ravi K. , and Mukesh Jain . 2012. “NGS QC Toolkit: A Toolkit for Quality Control of Next Generation Sequencing Data.” PLoS ONE 7: e30619. 10.1371/journal.pone.0030619 22312429 PMC3270013

[32] Masella, Andre P. , Andrea K. Bartram , Jakub M. Truszkowski , Daniel G. Brown , and Josh D. Neufeld . 2012. “PANDAseq: Paired‐End Assembler for Illumina Sequences.” BMC Bioinformatics 13: 31. 10.1186/1471-2105-13-31 22333067 PMC3471323

[33] Asnicar, Francesco , George Weingart , Timothy L. Tickle , Curtis Huttenhower , and Nicola Segata . 2015. “Compact Graphical Representation of Phylogenetic Data and Metadata with GraPhlAn.” PeerJ 3: e1029. 10.7717/peerj.1029 26157614 PMC4476132

[34] Shannon, Paul , Andrew Markiel , Owen Ozier , Nitin S. Baliga , Jonathan T. Wang , Daniel Ramage , Nada Amin , Benno Schwikowski , and Trey Ideker . 2003. “Cytoscape: A Software Environment for Integrated Models of Biomolecular Interaction Networks.” Genome Research 13: 2498–504. 10.1101/gr.1239303 14597658 PMC403769

[35] Wang, Jingzhong , and Hongjuan Jia . 2017. “Sediment Record of Environmental Change at Lake Lop Nur (Xinjiang, NW China) from 13.0 to 5.6 cal ka BP.” Chinese Journal of Oceanology and Limnology 35: 1070–78. 10.1007/s00343-017-6079-4

[36] Wang, Jihe , Hongbo Yuan , Jinchun Zhang , Guozhong Zhang , Hujun Liu , and Kongtai Liao . 2008. “Composition and Geographical Elements of the Flora in Kumtag Desert.” Journal of Desert Research 5: 860–67. https://kns.cnki.net/kcms/detail/detail.aspx?FileName=ZGSS200805012%26DbName=CJFQ2008

[37] Zhao, Shuwen , and Ling Yan . 2008. “The Floristic Characteristics of Seed Plants in Allah Desert Region.” Journal of Arid Land Resources and Environment 11: 167–74. http://en.cnki.com.cn/Article_en/CJFDTOTAL-GHZH200811029.htm

[38] Ji, Yinqiu , Louise Ashton , Scott M. Pedley , David P. Edwards , Yong Tang , Akihiro Nakamura , Roger Kitching , et al. 2013. “Reliable, Verifiable and Efficient Monitoring of Biodiversity Via Metabarcoding.” Ecology Letters 16: 1245–57. 10.1111/ele.12162 23910579

[39] Siefert, Andrew , Catherine Ravenscroft , David Althoff , Juan C. Alvarez‐Yépiz , Benjamin E. Carter , Kelsey L. Glennon , J. Mason Heberling , et al. 2012. “Scale Dependence of Vegetation–Environment Relationships: A Meta‐Analysis of Multivariate Data.” Journal of Vegetation Science 23: 942–51. 10.1111/j.1654-1103.2012.01401.x

[40] Van Couwenberghe, Rosalinde , Catherine Collet , Eric Lacombe , Jean‐Claude Pierrat , and Jean‐Claude Gégout . 2010. “Gap Partitioning Among Temperate Tree Species Across a Regional Soil Gradient in Windstorm‐Disturbed Forests.” Forest Ecology and Management 260: 146–54. 10.1016/j.foreco.2010.04.013

[41] Hawkins, Bradford A. , and Eric E. Porter . 2003. “Water–Energy Balance and the Geographic Pattern of Species Richness of Western Palearctic Butterflies.” Ecological Entomology 28: 678–86. 10.1111/j.1365-2311.2003.00551.x

[42] O'Brien, Eileen M. , Richard Field , and Robert J. Whittaker . 2000. “Climatic Gradients in Woody Plant (Tree and Shrub) Diversity: Water‐Energy Dynamics, Residual Variation, and Topography.” Oikos 89: 588–600. 10.1034/j.1600-0706.2000.890319.x

[43] Feng, Feng . 2008. “Spatial Patterns of Species Diversity of Seed Plants in China and Their Climatic Explanation.” Biodiversity Science 16: 470. 10.3724/SP.J.1003.2008.08027

[44] May, Robert M . 1988. “How Many Species Are There on Earth.” Science 241: 1441–49. 10.1126/science.241.4872.1441 17790039

[45] Zhao, Wenzhi , Xueli Chang , and Zhibin He . 2004. “Responses of Distribution Pattern of Desert Riparian Forests to Hydrologic Process in Ejina Oasis.” Science in China Series D: Earth Sciences 47: 21–31. 10.1360/04zd0003

[46] Fan, Zili , Yingjie Ma , Hong Zhang , Ranghui Wang , Yuanjie Zhao , and Hongfei Zhou . 2004. “Research of Eco–Water Table and Rational Depth of Groundwater of Tarim River Drainage Basin.” Arid Land Geography 27: 8–13. http://en.cnki.com.cn/Article_en/CJFDTOTAL-GHDL200401001.htm

[47] O'Brien, Eileen . 1998. “Water‐Energy Dynamics, Climate, and Prediction of Woody Plant Species Richness: An Interim General Model.” Journal of Biogeography 25: 379–98. 10.1046/j.1365-2699.1998.252166.x

[48] Zeng, Yanjun , Yanrong Wang , Zhibiao Nan , Dong Wei , Shanke Chen , and Baoer Li . 2003. “Soil Seed Banks of Different Grassland Types of Alashan Arid Desert Region, Inner Mongolia.” Chinese Journal of Applied Ecology 14: 1457–63. http://en.cnki.com.cn/Article_en/CJFDTotal-YYSB200309010.htm 14732998

[49] Wang, Cuihong . 2004. *Research on the Biodiversity Distribution Patterns in Mainland of China*. Taiyuan, Shanxi: Shanxi University. http://cdmd.cnki.com.cn/Article/CDMD-10108-2004077177.htm

[50] Yong, Shipeng , and Zhongyuan Zhu . 1999. “A Fundamental Characteristics of Gobi Desert Flora in the Central Asia.” Acta Scientiarum Naturalium Universitatis Inner Mongolia 21: 214–17. http://en.cnki.com.cn/Article_en/CJFDTOTAL-NMGX199002014.htm

[51] Jiao, Shuo , Haiyan Chu , Baogang Zhang , Xiaorong Wei , Weimin Chen , and Gehong Wei . 2022. “Linking Soil Fungi to Bacterial Community Assembly in Arid Ecosystems.” iMeta 1: e2. 10.1002/imt2.2 PMC10989902 38867731

