# Supplementary material for: What determines plant species diversity along the Modern Silk Road in the east?
Source: Imeta. 2023 Jan 9;2(1):e74. doi: 10.1002/imt2.74 (PMC10989921; doi:10.1002/imt2.74)
Supplement: Supplementary file 1 — Supporting Information [file IMT2-2-e74-s001.docx]

**Supporting Figures in this study**


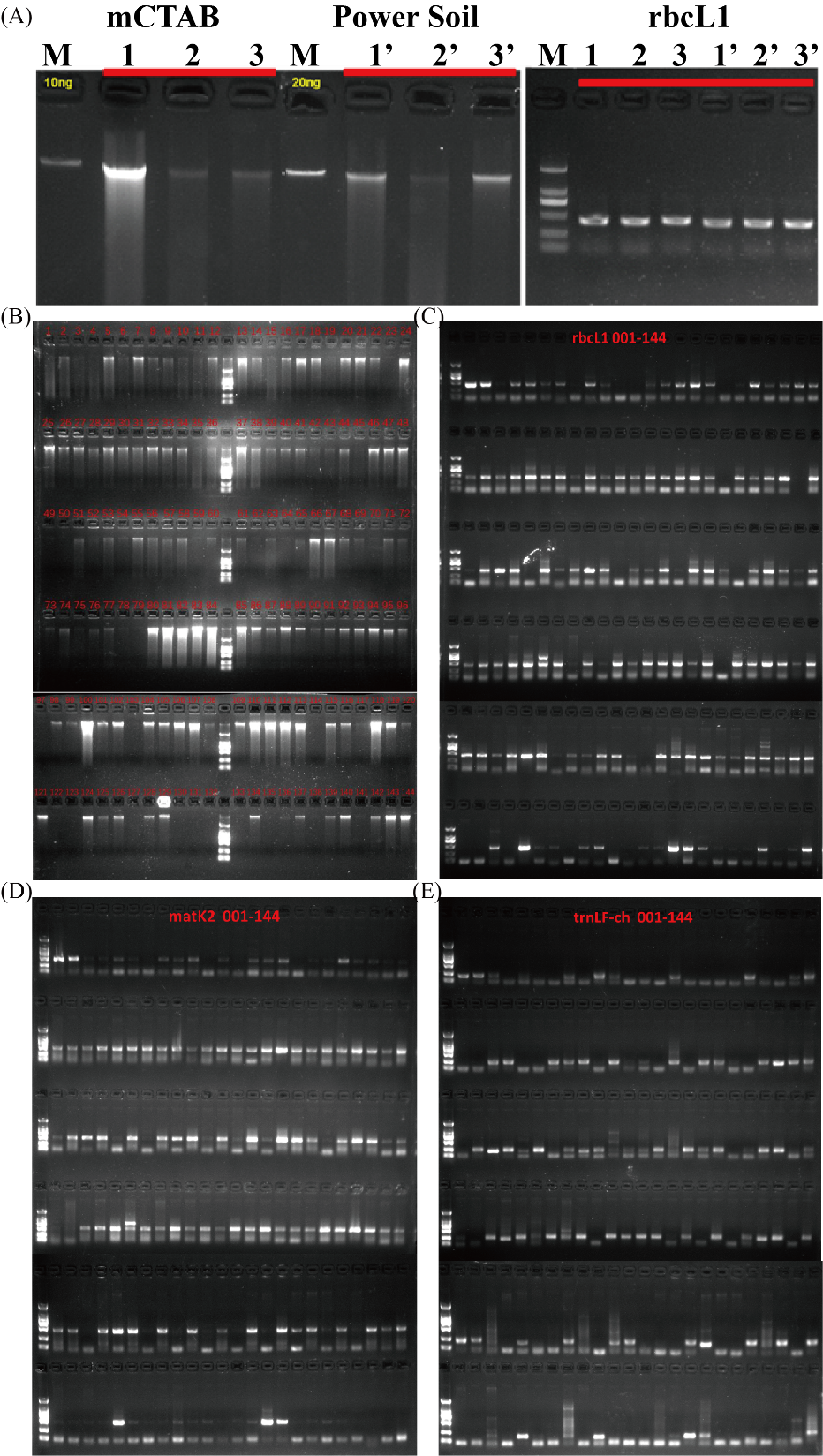


**Figure S1.** Feasibility validation of Power Soil DNA extraction kit and DNA, PCR results of each sample. (A): Comparison of Power Soil DNA extraction kit and mCTAB method; (B): DNA situation obtained from topsoil samples using Power Soil DNA extraction kit; (C): rbcL1 PCR amplification situation in each sample; (D): matK2 PCR amplification situation in each sample; (E): trnL-intron PCR amplification situation in each sample. The sample distribution of (C), (D) and, (E) is the same as that of (B).

**
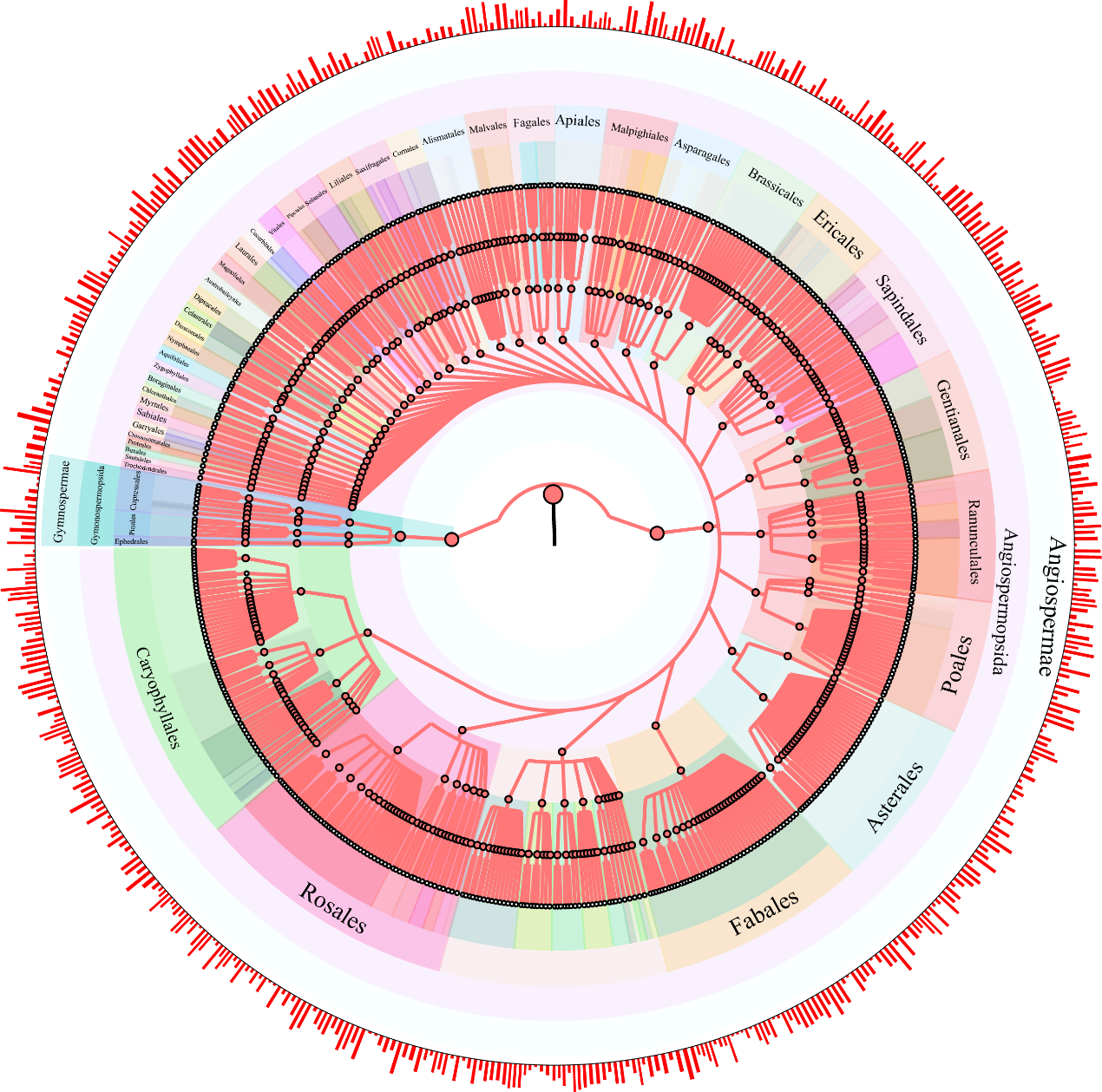
**

**Figure S2.** General situation of wild plant species in desert area of northwest China. Statistics of relative species richness were presented in the outside ring(red), the value is the logarithm of sequence number based on 10 in each species.

**
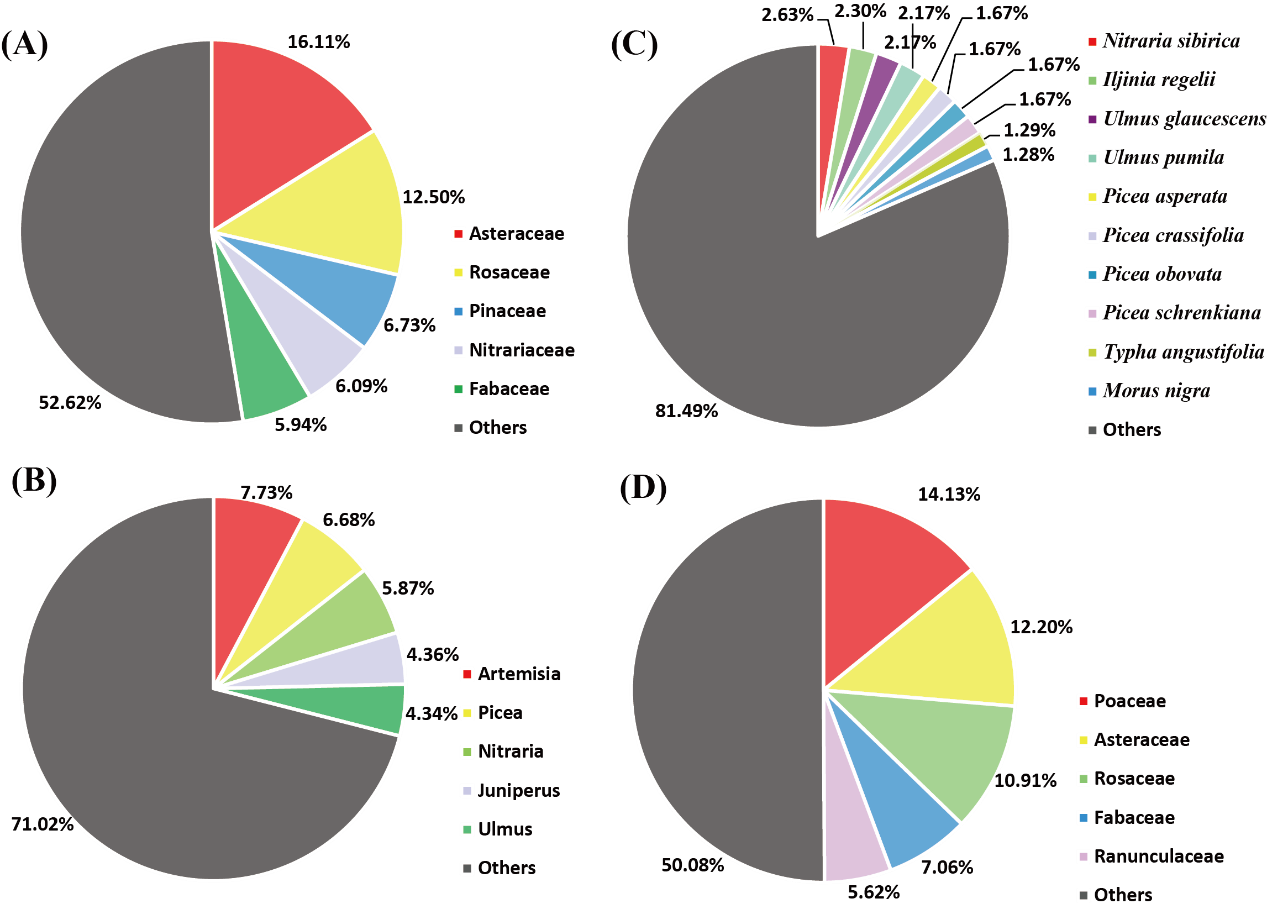
**

**Figure S3.** Majority plant groups in the 144 soil samples. (A): Top five families based on relative abundance in wild species; (B): Top five genera based on relative abundance in wild species; (C): Top ten species based on relative abundance in wild species; (D): Top five families based on species number in wild species.

**
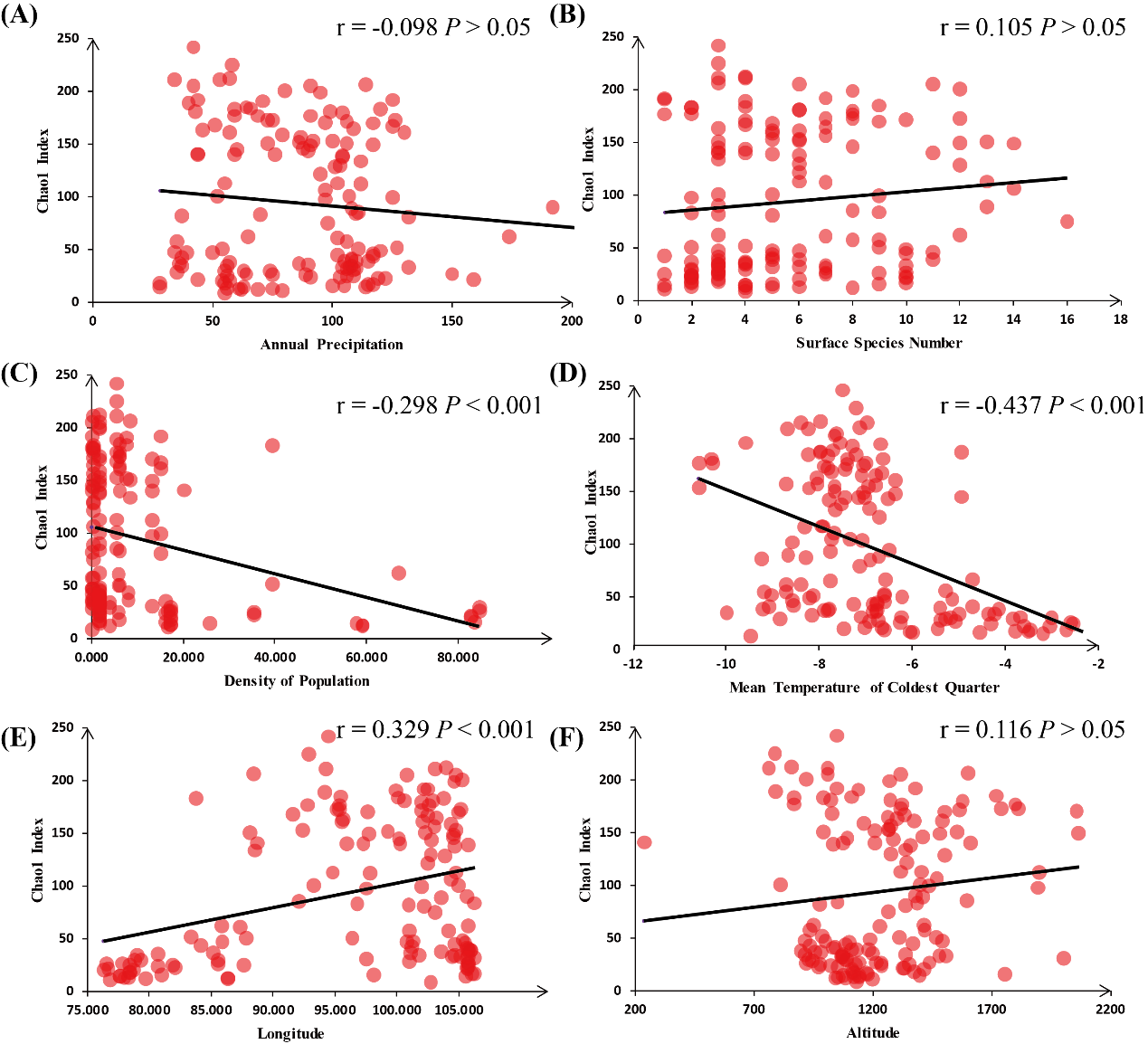
**

**Figure S4.** Correlation analysis between Chao1 Index and potential affecting factors. Chao1 Index correlation results with (A): Annual Precipitation; (B): Surface Species Number; (C): Density of Population; (D): Mean Temperature of Coldest Quarter; (E): Longitude; (F): Altitude.
